# Low-density pedicle screw in adolescent idiopathic scoliosis: a systematic review and meta-analysis of 1,762 patients

**DOI:** 10.3389/fsurg.2025.1607323

**Published:** 2025-07-30

**Authors:** Bin Zheng, Qiang Zhou, Xuanwen Liu, Ke Ma, Zhe Qiang

**Affiliations:** ^1^ Spine Surgery, Peking University People’s Hospital, Beijing, China; ^2^ Department of Orthopedic Surgery, 363 Hospital, Chengdu, Sichuan, China; ^3^ Orthopedics Department, Huailai County Hospital, Zhangjiakou, Hebei, China

**Keywords:** adolescent idiopathic scoliosis, screw density, high density, low density, deformity

## Abstract

**Background:**

High-density pedicle screws provide satisfactory correction in adolescent idiopathic scoliosis (AIS) but add to the operative time, blood loss, and cost; low-density constructs may mitigate these burdens and achieve similar correction results. Studies use inconsistent density cutoffs (most often <1.6 screws/level) and report conflicting results; therefore, we performed a systematic review and meta-analysis to clarify the clinical, radiographic, and economic impact of low screw density in patients with AIS.

**Methods:**

A systematic review and meta-analysis were conducted following PRISMA guidelines. The PubMed, Web of Science, and Embase databases were searched until December 2024 for comparative studies. The outcomes analyzed included surgical and safety parameters (blood loss, operative time, revision rates, and complications), radiographic outcomes (Cobb angle, correction rate, and thoracic kyphosis), and implant costs. Statistical analyses were performed using RevMan 5.4, with fixed- or random-effects models applied on the basis of heterogeneity (*I*² threshold < 50%).

**Results:**

Twenty-one studies comprising 1,762 patients met the inclusion criteria. Low-density screws were superior in reducing blood loss [mean difference (MD) = −88.06, *P* = 0.01] and operative time (MD = −22.27, *P* = 0.02), with no significant difference in revision rates (*P* = 0.78) or complications (*P* = 0.64). No differences were observed between the groups in the final Cobb angle (*P* = 0.4), Cobb correction rate (*P* = 0.21), or thoracic kyphosis (*P* = 0.43). The per-level implant cost was lower (standard mean difference = −1.32, *P* < 0.00001) in the low-density group.

**Conclusion:**

Compared with high-density screws, low-density pedicle screws provide comparable radiographic and safety outcomes while reducing the operative time, blood loss, and cost. These findings support the use of low-density constructs in AIS surgery, although the variability in study designs and screw density definitions warrants further research. Future multicenter randomized controlled trials are needed to refine the optimal screw density strategies for treating AIS.

**Systematic Review Registration:**

https://www.crd.york.ac.uk/PROSPERO/view/CRD420251088403, PROSPERO CRD420251088403.

## 1 Introduction

Adolescent idiopathic scoliosis (AIS) is a complex three-dimensional spinal deformity (1, 2). Studies have reported that compared with other instruments, pedicle-screw constructs provide enhanced three-dimensional deformity correction, achieve satisfactory three-column fixation, and minimize neurological complications (3–6). Although pedicle-screw constructs provide strong fixation and correction, excessive instrumentation may sacrifice more motion segments than necessary. Optimizing the fusion level and screw density may help limit the fused area and preserve adjacent segment mobility. Moreover, the high cost of AIS surgery is closely related to the use of pedicle screws, which account for a significant proportion of hospital expenses (7, 8). The use of fewer pedicle screws has been shown to reduce hospital costs and decrease the risk of neurological complications (9–11).

Given the cost of pedicle screws, reducing the number of screws improves surgical efficiency and potentially reduces the costs associated with spinal instrumentation. Compared with high-density constructs, low-density pedicle-screw constructions result in shorter operative times and less blood loss (12, 13). The reported cutoffs for low-density screws range from 1.0 to 1.6 screws/level, whereas those for high-density screws range from 1.3 to 2.0 screws/level, creating overlap between categories. Gotfryd and Avanzi (14) reported that there was no significant difference in radiographic corrective outcomes between patients with a density > 1.6 and those with a density < 1.2 (14). Chang et al. (15) reported similar radiographic outcomes between a density of 1.9 and a density of 1.1 (15). However, Ketenci et al. (16) reported that a density of 2 was superior to a density of 1.14 in terms of radiographic outcomes (16). A comprehensive review comparing the efficacy and safety of low- vs. high-density pedicle screws is still lacking.

The aim of this study is to evaluate the efficacy and safety of low-density and high-density pedicle screws in adolescent scoliosis patients by performing a systematic review and meta-analysis of published studies.

## 2 Methods

Our methodology and reporting of this systematic review followed PRISMA guidelines (17). Systematic review protocol is registered in PROSPERO (CRD420251088403).

### 2.1 Search strategy

To collect comprehensive published studies, we used the search terms ((pedicle screw density) OR (consecutive pedicle screw) OR (interval pedicle screw)) AND (adolescent idiopathic scoliosis) in PubMed, Web of Science, and Embase, covering the period from the inception of each database to December 2024. Two authors also screened reference lists for additional studies.

### 2.2 Inclusion criteria

Eligibility criteria were established *a priori* with the population, intervention, comparison, outcome, study design (PICOS) framework. We included studies—randomized controlled trials (RCTs) and prospective or retrospective cohort studies—that examined surgical treatment of AIS across all Lenke classifications. Eligible studies compared low-density pedicle-screw constructs (<1.6 screws per fused level) with high-density constructs (>1.6 screws per level). We excluded studies on congenital, degenerative, neuromuscular, or neurofibromatosis-associated scoliosis, as well as single-case reports and technical notes. Studies were included if they reported at least one prespecified outcome: intraoperative blood loss, operative time, revision rate, cost per fused level, complication rate, major curve magnitude, thoracic kyphosis, or major curve correction rate—defined as (preoperative Cobb angle − postoperative Cobb angle)/preoperative Cobb angle × 100%. Two reviewers independently screened titles and abstracts retrieved from the search and then assessed the full texts of potentially relevant articles; any disagreements were resolved through discussion and consensus.

### 2.3 Study selection

Two reviewers independently conducted the study selection in EndNote 20 (Clarivate Analytics, Philadelphia, PA, USA), a reference-management platform that automates duplicate removal and facilitates screening. The screening proceeded in three sequential stages—titles, abstracts, and full texts (18). Any disagreements that arose at any stage were resolved through discussion with the corresponding author until consensus was achieved.

### 2.4 Risk of bias

For observational studies, two authors independently assessed risk on the basis of the Newcastle–Ottawa scale (NOS) (19). For RCTs, both authors used the Cochrane recommended criteria (20). Following the PRISMA and Cochrane Collaboration criteria, two authors independently assessed the risk of bias in the RCTs in the following areas: (1) random sequence generation, (2) allocation concealment, (3) blinding of participants and personnel, (4) blinding of outcome assessment, (5) incomplete outcome data, (6) selective reporting, and (7) other biases.

### 2.5 Statistical analysis

The authors performed the analysis with RevMan 5.4 software (The Cochrane Collaboration, Copenhagen, Denmark). The software assessed heterogeneity via the *χ*^2^ test and inconsistency index statistics (*I*^2^). When significant heterogeneity emerged (*I*^2^ > 50%), the analysis adopted a random-effects model. Conversely, in cases of homogeneity (*I*^2^ ≤ 50%), a fixed-effects model was used. For effect analysis, the mean difference (MD) and odds ratio (OR) served as the statistical measures for continuous and binary variables, respectively, each accompanied by a 95% confidence interval (CI). In the included studies, the cost of the currency was different. Therefore, in the cost analysis, the standard mean difference (SMD) and 95% CI were applied for analysis.

## 3 Results

### 3.1 Summary of the included studies

**Figure 1:**
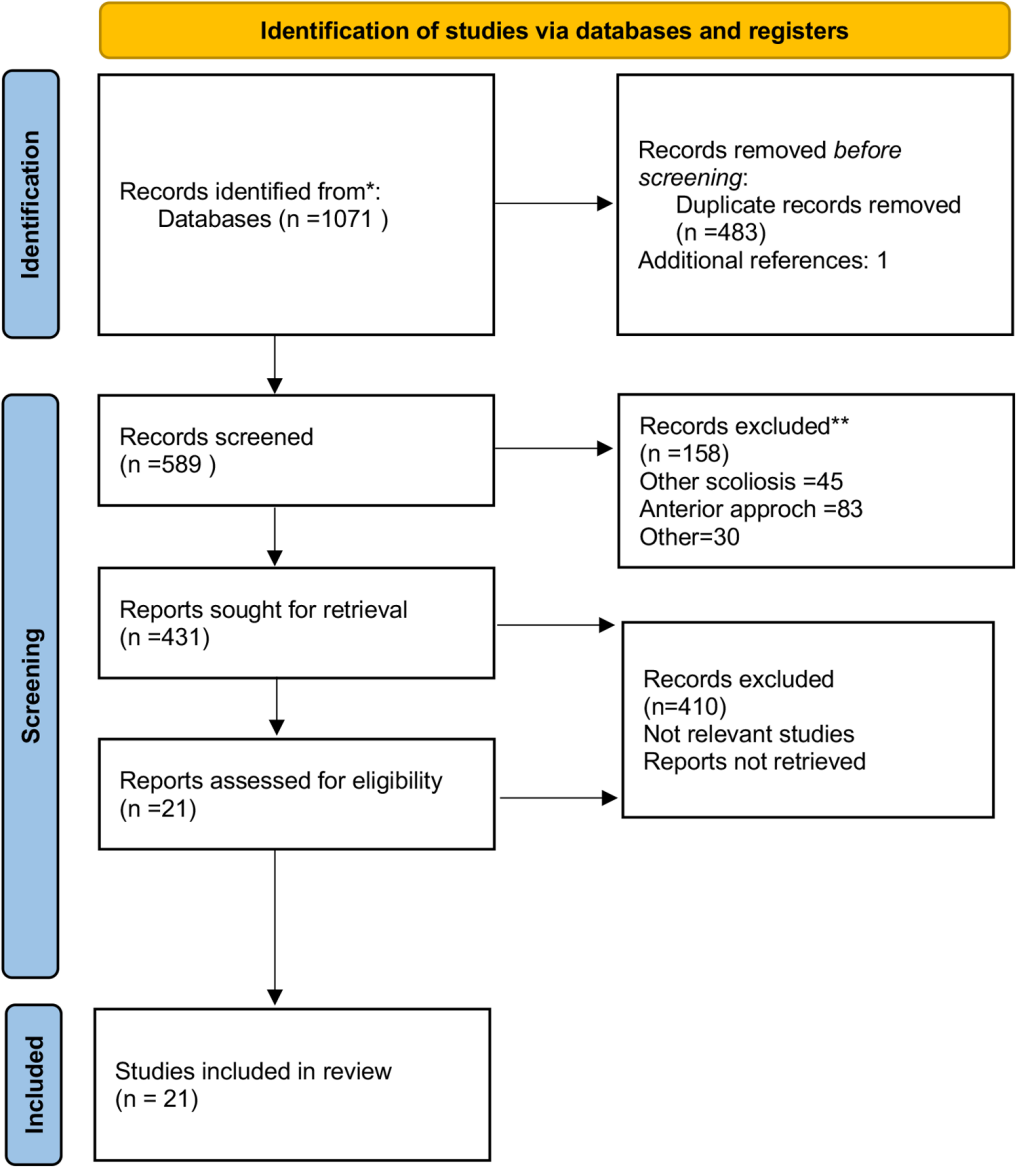
Flowchart of study selection.

After screening 1,072 studies, 21 studies (12, 14–16, 21–37) including 1,762 patients were eligible for meta-analysis. The selection process is shown in Figure 1. A total of 884 patients were in the low-density group, 91 were in the medium-density group, and 787 were in the high-density group. Three studies were RCTs (14, 26, 37), and the other 18 were retrospective studies.

The included studies used different thresholds for defining pedicle-screw density. Low density was generally set at ≤1.0–1.6 screws per fused level (with most papers clustering ∼1.2–1.4 screws/level), whereas high density was defined as >1.4 up to ≥2.0 screws/level. Some studies also introduced an intermediate “medium-density” category. Although the cutoffs varied, this meta-analysis retained the original definitions of each study when classifying cases into low- and high-density groups. The characteristics of the included studies are given in Table 1.

**Table 1:** Characteristic of included studies.

Study	Lenke I/II/III/IV/V/VI	Sample size			Definition density			Actual density			Complications		
		Low	Medium	High	Low	Medium	High	Low	Medium	High	Low	Medium	High
Li et al. (26)	L: 15/0/0/0/0/0 H: 15/0/0/0/0/0	15		15	—		—	—		—	Pseudarthrosis with instrumentation failure (*n* = 1) Supeficial wound infection (*n* = 1)		Deep wound infection (*n* = 2)
Tao et al. (33)	29/15/13/0/20/0	24		22	—		—	—		—	—	—	—
Tsirikos and Subramanian (34)	L: 44/12/49/9/32/15 H: 15/4/18/5/3/6	161		51	—		—	1.38 (1.2–1.8)		2	Deep wound infection (*n* = 1) Superficial wound infection (*n* = 1) Transient brachial plexus neuropraxia (*n* = 1) Superior mesenteric artery syndrome (*n* = 1)		Transient loss of intraoperative spinal cord monitoring traces/no neurological deficit (*n* = 1) Deep wound infection (*n* = 1) Prominent instrumentation (*n* = 1)
Gotfryd and Avanzi (14)	L: 23/0/0/0/0/0 H: 23/0/0/0/0/0	23		23				59.9%		80.3%	—		Non-infected operative wound seroma (*n* = 1) 3-cm decompensation of the trunk to the left side (*n* = 1)
Bharucha et al. (22)	L: 57/0/0/0/0/0 H: 34/0/0/0/0/0	57		34				1.1 ± 0.1		1.6 ± 0.2	Misplaced L1 pedicle screw causing radicular pain (*n* = 1) Loss of fixation of a proximal end instrumented vertebrae (*n* = 1) Dissociation of the rod from the pedicle screw (*n* = 1)		—
Liu et al. (27)		77			<1.2		≥1.2				—		—
A	L: 18/0/0/0/0/0 H: 17/0/0/0/0/0	18		17	<1.2		≥1.2				—		—
B	L: 22/0/0/0/0/0 H: 20/0/0/0/0/0	22		20	<1.2		≥1.2				—		—
Morr et al. (29)	L: 20/0/0/0/0/0 H: 20/0/0/0/0/0	20		20	—		—	—		—	—		—
Wang et al. (35)	L: 20/0/0/0/0/0 H: 16/0/0/0/0/0	20		20	—		—	—		—	—		Wound infection (*n* = 1)
Kemppainen et al. (24)	L: 17/6/2/0/0/1 H: 17/3/0/1/0/5	26		26	—		—	1.1 ± 0.06		1.5 ± 0.22	Supeficial wound dehiscence (*n* = 1) Deep wound infection (*n* = 1) Unilateral broken pedicle screw at the lowest instrumented vertebrae (*n* = 1)		Proximal junctional kyphosis (*n* = 1) Metallosis at lowest instrumented vertebrae (*n* = 1) Deep wound infection (*n* = 1)
Ketenci et al. (16)	L: 38/0/0/0/0/0 H: 38/0/0/0/0/0	38		38	—		—	1.14 ± 0.24		2	—		—
Shen et al. (32)	L: 28/0/0/0/0/0 H: 34/0/0/0/0/0	28		34	<1.61		>1.61	1.3 ± 0.2		1.83 ± 0.10	—		Misplaced T9 pedicle screw causing neurologic symptoms (*n* = 1)
Luo et al. (28)	L: 25/0/0/0/0/0 H: 22/0/0/0/0/0	25	18	22	—	—	—	1.3 ± 0.2	1.6 ± 0.1	2.0 ± 0.0	Poor wound healing (*n* = 1)		Adding-on phenomena (*n* = 1) Superficial wound infection (*n* = 1)
Sariyilmaz et al. (30)	L: 0/0/0/0/28/0 H: 0/0/0/0/31/0	28		31	<1.6		≥1.6	75.4%		96.6%	—		—
Yeh et al. (36)	59/19/12/6/22/9	16	73	38	≤1.4		>1.7	1.31 ± 0.07	1.55 ± 0.08	1.83 ± 0.10	—	—	—
Şenköylü et al. (31)	24/6/14/6/6/4	30		30	—		—	—		—	—		
Ferlic et al. (12)	L: 22/4/0/0/0/0 H: 22/3/0/0/0/0	26		25	—		—	1.5 ± 0.1		1.9 ± 0.1	An accidental durotomy with drainage of cerebrospinal fluid (*n* = 1) An anterior breach of a concave-side screw (*n* = 1) A screw loosened in the lowest instrumented vertebra (*n* = 1)		An anterior breach of a concave-side screw (*n* = 1)
Lertudomphonwanit et al. (25)	99/23/0/0/0/0	57		65	<1.5		≥1.5	1.3 ± 0.1		1.7 ± 0.1	Adding-on phenomena (*n* = 1)		Adding-on phenomena (*n* = 2)
Chotigavanichaya et al. (23)	L: 18/4/5/0/17/2 H: 29/3/2/0/33/9	46		76	1.1–1.4		≥1.4	1.3 ± 0.1		1.7 ± 0.2	—		—
Chang et al. (15)	L: 53/0/0/0/0/0 H: 19/0/0/0/0/0	53		19	—		—	1.0 ± 0.1		1.9 ± 0.2	Adding-on phenomena (*n* = 6) Coronal decompensation (*n* = 3)		Adding-on phenomena (*n* = 1) Coronal decompensation (*n* = 1)
Baymurat et al. (21)	L: 38/14/0/0/0/0 H: 37/17/0/0/0/0	52		54	—		—	—	—	—	—		—
Larson et al. (37)	L: 99/0/0/0/0/0 H: 107/0/0/0/0/0	108		103	≤1.4		>1.4				Pedicle screw displacement (*n* = 1) Infection (*n* = 4)		Implant failure (*n* = 1) Infection (*n* = 5)

**Figure 2:**
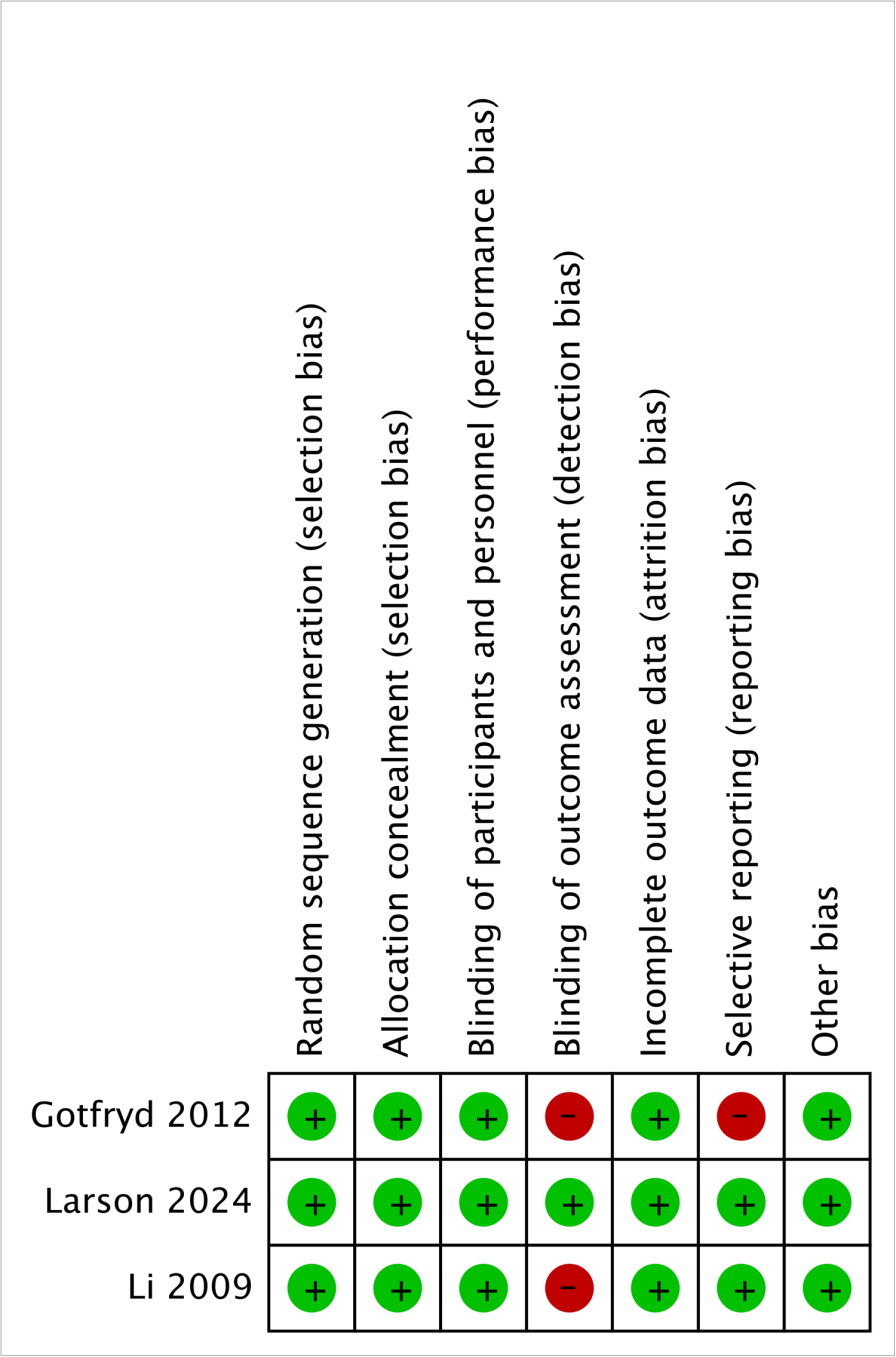
Risk of bias of the RCTs.

Figure 2 shows that the three included RCTs were evaluated at a low risk of bias for random sequence generation, allocation concealment, performance bias, attrition bias, and other domains; only outcome-assessor blinding and selective reporting displayed isolated high-risk ratings, so their overall methodological quality remained moderate to high. Table 2 shows that, among the 18 cohort studies, 11 earned eight NOS stars, 4 earned seven, and 3 earned six, with none rated ≤5; most point deductions stemmed from follow-up adequacy. Taken together, the available literature provided moderate-to high-quality evidence to support the conclusions of the review, despite some limitations in terms of blinding and follow-up.

**Table 2:** Risk of bias assessment using the Newcastle–Ottawa scale for observational studies.

Study	Selection				Comparability		Outcome			Total scores
	Exposed cohort	Non-exposed cohort	Ascertainment of exposure	Outcome of interest	The most important factor	Additional factor	Assessment of outcomes	Length of follow-up	Adequacy of follow-up	
Tao et al. (33)	★	★	☆	★	★	★	☆	★	★	7
Tsirikos and Subramanian (34)	★	★	★	★	★	★	☆	★	★	8
Bharucha et al. (22)	★	★	★	★	★	★	☆	★	★	8
Liu et al. (27)	★	★	★	★	★	★	☆	☆	☆	6
Morr et al. (29)	★	★	☆	★	★	★	☆	★	★	7
Wang et al. (35)	★	★	☆	★	★	★	★	☆	☆	6
Kemppainen et al. (24)	★	★	★	★	★	★	☆	★	★	8
Ketenci et al. (16)	★	★	★	★	★	★	☆	☆	☆	6
Shen et al. (32)	★	★	★	★	★	★	☆	★	★	8
Luo et al. (28)	★	★	★	★	★	★	☆	★	★	8
Sariyilmaz et al. (30)	★	★	★	★	★	★	☆	★	★	8
Yeh et al. (36)	★	★	★	★	★	★	☆	★	★	8
Şenköylü et al. (31)	★	★	☆	★	★	★	☆	★	★	7
Ferlic et al. (12)	★	★	★	★	★	★	☆	★	★	8
Lertudomphonwanit et al. (25)	★	★	★	★	★	★	☆	★	★	8
Chotigavanichaya et al. (23)	★	★	★	★	★	★	☆	★	★	8
Chang et al. (15)	★	★	★	★	★	★	☆	★	★	8
Baymurat et al. (21)	★	★	☆	★	★	★	☆	★	★	7

### 3.2 Surgical and safety parameters

**Figure 3:**
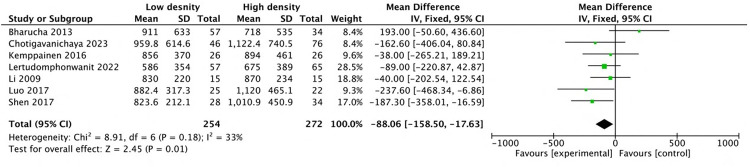
Comparison of blood loss in the low-density and high-density groups.

**Figure 4:**
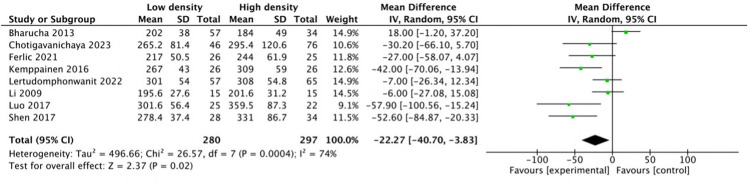
Comparison of the operative time between the low-density and high-density groups.

**Figure 5:**
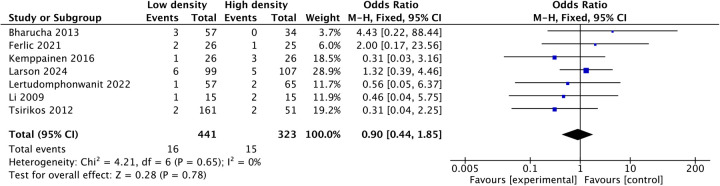
Comparison of revision surgeries in the low-density and high-density groups.

**Figure 6:**
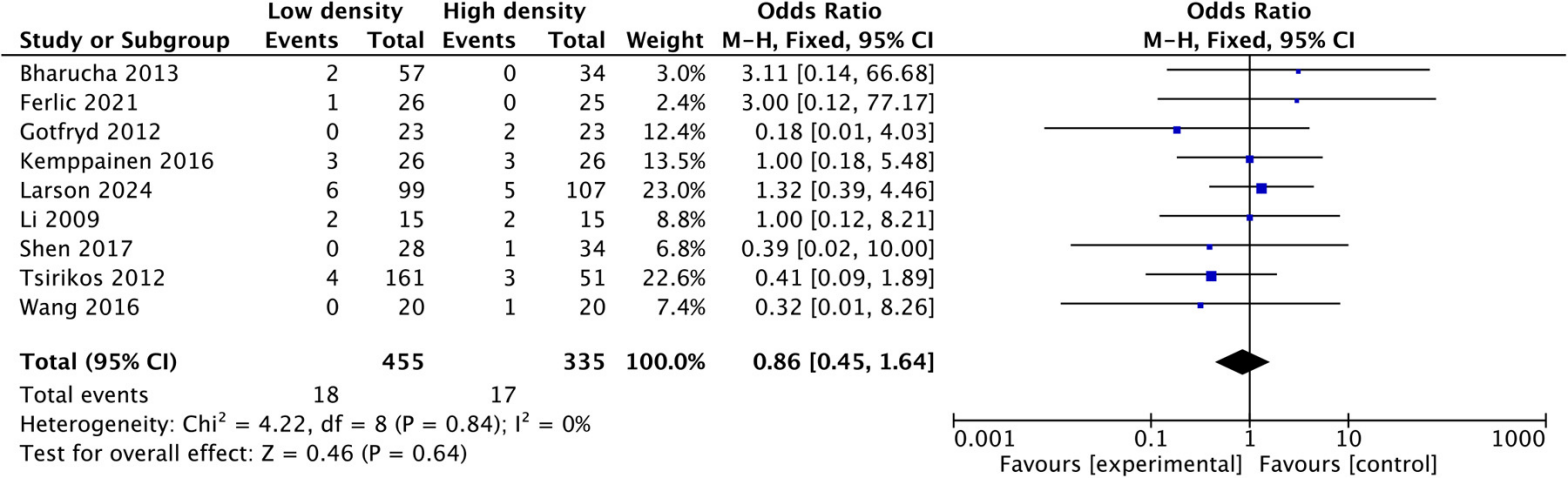
Comparison of complications between the low-density and high-density groups.

Pooled analysis of seven studies (21, 23–26, 28, 32) revealed that the mean intraoperative blood loss in the low-density group was reduced by 88.06 mL (MD: −88.06; 95% CI: −158.5 to −17.6; *P* = 0.01; *I*^2^ = 33%), as shown in Figure 3. Three studies (24, 28, 32) reported shorter operative times in the low-density group, and five studies (12, 22, 23, 25, 26) reported similar operative times between the two groups. Pooled analysis of eight studies revealed that the operative time decreased by 22.27 min (MD: −22.27, 95% CI: −40.7 to −3.83, *P* = 0.02; heterogeneity Chi^2^ = 26.57, df = 7, *P* = 0.0004, *I*^2^ = 74%) in the low-density group, as shown in Figure 4. These gains in surgical efficiency were not offset by increased risk. A pooled analysis of seven studies (12, 22, 24–26, 34, 37) reported similar revision rates (*P* = 0.78), as shown in Figure 5. The pooled analysis of nine studies (12, 14, 22, 24, 26, 32, 34, 35, 37) revealed similar complication events (*P* = 0.64), as shown in Figure 6.

### 3.3 Deformity correction

**Figure 7:**
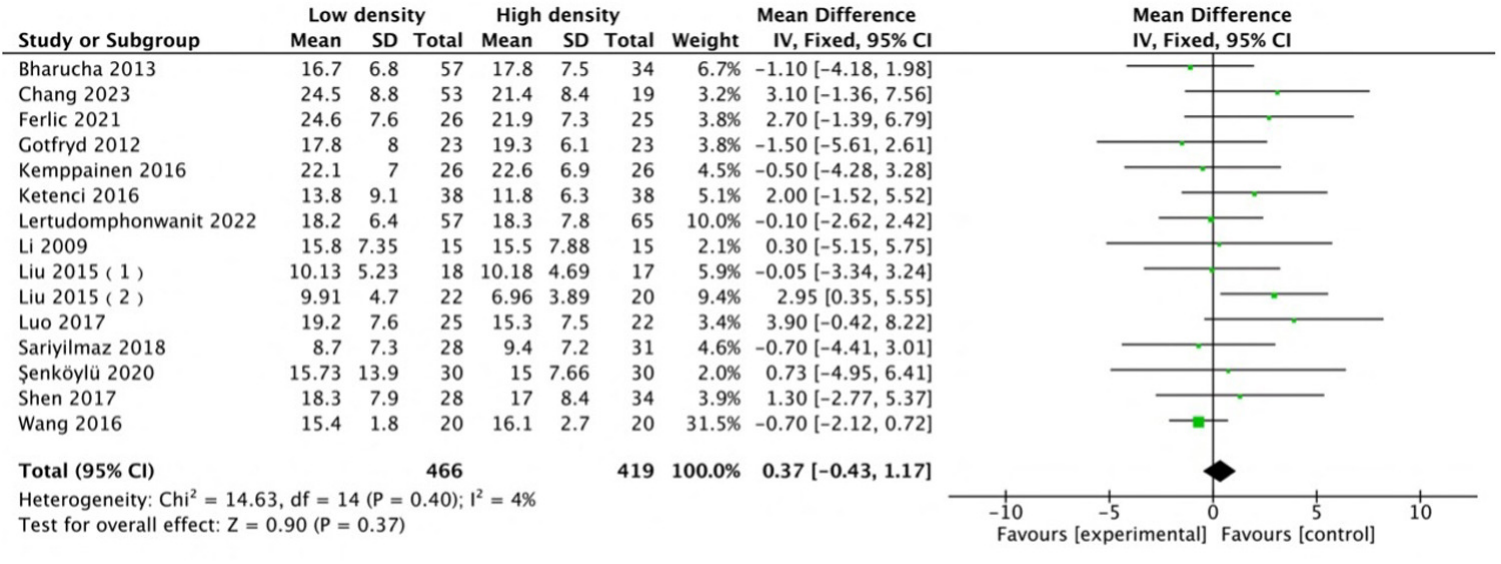
Comparison of the Cobb angle between the low-density and high-density groups.

**Figure 8:**
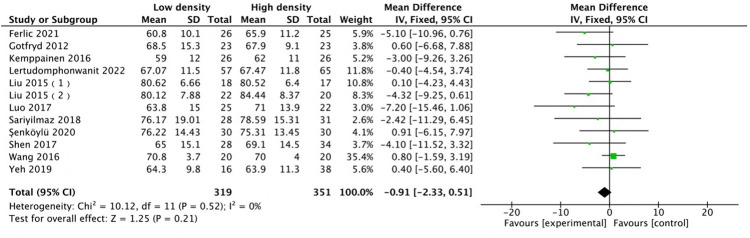
Comparison of major curves of correction in the low-density and high-density groups.

**Figure 9:**
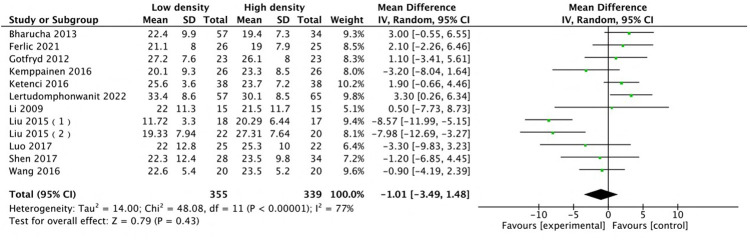
Comparison of thoracic kyphosis between the low-density and high-density groups.

The radiographic results indicated that the two groups were comparable in terms of deformity correction. Among all the included studies, 14 studies (12, 14–16, 22, 24–28, 30–32, 35) with 15 comparison groups were pooled for major Cobb analysis, and the results indicated no difference (MD: 0.37, 95% CI: −0.43 to 1.17, *P* = 0.37; heterogeneity Chi^2^ = 14.63, df = 14, *P* = 0.4, *I*^2^ = 4%), as shown in Figure 7. A pooled analysis of the studies (12, 14, 24, 25, 27, 28, 30–32, 35–37) revealed no difference in major curve correction (MD: −0.91, 95% CI: −2.33 to 0.51, *P* = 0.21; heterogeneity Chi^2^ = 10.21, df = 11, *P* = 0.52, *I*^2^ = 0%), as shown in Figure 8. With respect to thoracic kyphosis, Liu et al. (27) reported less thoracic kyphosis at the final follow-up in the low-density group. Lertudomphonwanit et al. (25) reported greater thoracic kyphosis. Other studies (12, 14, 16, 22, 24–28, 32, 35) reported no difference between the two groups. Pooled analysis revealed no difference (MD: −1.01, 95% CI: −3.49 to 1.48, *P* = 0.43; heterogeneity Chi^2^ = 48.08, df = 11, *P* < 0.00001, *I*^2^ = 77%), as shown in Figure 9.

### 3.4 Implant cost per level

**Figure 10:**
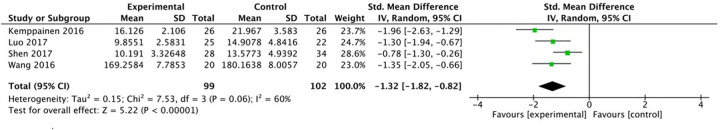
Comparison of cost per level in the low-density and high-density groups.

Four studies (24, 28, 32, 38) reported a lower cost per level in the low-density group, and pooled analysis revealed a lower cost (SMD: −1.32, 95% CI: −1.82 to −0.82, *P* < 0.00001; heterogeneity Chi^2^ = 7.53, df = 3, *P* = 0.06, *I*^2^ = 60%) in the low-density group, as shown in Figure 10.

## 4 Discussion

Pedicle-screw constructs have become the standard instrumentation in the treatment of AIS. Because of the three-column anchorage of pedicle screws, more corrective forces can be applied, thus allowing three-dimensional correction of the curve. Despite advancements in pedicle screw techniques and the use of neuromonitoring, surgeons continue to encounter challenges such as neurologic complications, high intraoperative blood loss, and elevated implant costs when high-density screw constructs are used (39–42).

The pedicle screw density is defined as the number of pedicle screws per level of spinal fusion. If low-density constructs prove as efficacious as high-density constructs in correcting the spinal curvature, they could improve surgical efficiency while also significantly reducing implantation costs. Previous studies have not been consistent in their results on the corrective ability of high-density vs. low-density pedicle-screw constructs in AIS (14, 32, 35). Therefore, in this study, we systematically reviewed the literature and performed a meta-analysis to compare low- and high-density screw constructs.

The studies analyzed included AIS patients with mixed Lenke curve types. For completeness, we pooled data from all eligible studies, and the heterogeneity of the outcomes was acceptable. This suggests that the study results were comparable across the included papers.

In clinical studies, low density was superior in reducing blood loss and operative time. These improvements were an expected result of placing fewer screws. Although the differences in blood loss and operative time were statistically significant, their clinical significance needs further evaluation. The pooled analysis revealed that the low-density group had an average blood loss reduction of 88.06 mL, which corresponds to approximately 9.7% of the average intraoperative blood loss in the high-density group. This relative reduction, while modest in absolute terms, may be clinically meaningful in certain populations, such as those with comorbidities or increased bleeding risk. Similarly, the average reduction in operative time in the low-density group was 22.27 min, accounting for approximately 5.6% of the mean operative time in the high-density group. Although not large, a shorter surgical duration may help reduce anesthetic exposure and surgical team fatigue, potentially improving safety in elderly or high-risk patients. There was no difference between the two methods in terms of complications. Although the use of more pedicle screws could theoretically increase the risk of screw-related damage or prolong surgery, in the included studies, the surgeries were performed by experienced surgeons (minimizing any learning curve issues) (43). There was no difference in complications such as nerve damage or pedicle screw misplacement. The current study did not evaluate whether surgeon expertise influenced the results. This may represent a potential confounding factor, as operative experience can significantly impact complication rates, surgical precision, and operative efficiency.

Given the advantages of low-density screws in terms of a shorter operative time and less bleeding, the current controversy over low-density screws vs. high-density screws focuses on the ability of low-density screws to achieve comparable correction capacity and maintain long-term results. In this study, a meta-analysis of the radiographic parameters reported in the included studies was performed to obtain more plausible results in the form of an expanded sample size. There was no difference between low-density and high-density screws in terms of the final Cobb angle, Cobb angle correction, or final follow-up of thoracic kyphosis. In scoliosis surgery, screws provide the point at which the implanted rods are anchored. Studies have shown that the quality of the screw anchorage points is more important than the quantity (44). The stability of the screw as an anchoring point is critical. A strong anchorage point can more effectively carry and distribute the corrective forces applied to the spine. Even with many anchorage points, if these are not stable enough, it will be difficult to effectively correct scoliosis or maintain correction. Properly placed screws not only are more tightly integrated into the spine but also reduce the risk of bone damage and implant loosening (45, 46). Importantly, even a small number of screws, when properly placed, can achieve good orthopedic results. During scoliosis correction, a balanced distribution of forces is more crucial than the magnitude of any single force. High-quality anchorage points distribute corrective forces more efficiently, reducing the stress on any single segment (38, 44, 47).

Although implant density is an important factor in optimizing the outcomes and cost-effectiveness of surgery for AIS, it should not be considered in isolation. The extent of spinal fusion—particularly the upper instrumented vertebra (UIV) and lower instrumented vertebra (LIV)—plays a critical role in balancing deformity correction with postoperative mobility. Recent biomechanical studies have demonstrated that, compared with simply increasing the number of screws, the positioning of anchor points, construct configuration, and screw quality have a more significant impact on the load distribution and construct stability (48, 49). Computer-based biomechanical studies have also shown that individualized correction strategies and preoperative objectives influence radiographic outcomes more than implant density alone does (50). However, in this systematic review, most included studies did not consistently report UIV and LIV selections or stratify results on the basis of the fusion range, which represents a limitation. Therefore, future studies should comprehensively consider fusion level selection, construction strategies, and implant density to optimize both correction outcomes and functional preservation in AIS surgery.

The high cost of AIS surgery is closely related to the use of expensive pedicle screws, which account for a significant proportion of hospital expenses (8, 51). The use of fewer pedicle screws has been shown to reduce hospital costs and decrease the risk of neurological complications. Consistent with our findings, multiple studies reported lower costs with low-density constructs, and our meta-analysis confirmed this trend. Larson et al. (52) examined the NIS database for AIS cases in the US and reported that switching from high-density to low-density screw patterns would reduce total national costs by an estimated $11–$20 million annually (52).

The limitations of this study were as follows: (1) Most of the studies included were retrospective studies, and only two were high-quality RCTs. In addition, the sample size in two RCTs was limited (30 and 46 total patients). The bias introduced was unavoidable. (2) A major limitation of the study was the lack of uniform definitions for low- and high-density screw constructs across the included studies. This inconsistency may have introduced clinical heterogeneity, potentially masking subtle differences in correction capacity or complication profiles. While overall statistical heterogeneity was acceptable, future studies should adopt standardized density thresholds or explore the dose-dependent effects of screw number to clarify optimal construct strategies. (3) The included studies exhibited variability in curve classification and deformity severity. Although the meta-analysis demonstrated acceptable and comparable heterogeneity, the lack of uniformity in baseline characteristics limited the precision of pooled estimates. Future research should aim for more standardized study designs with consistent definitions of curve type and severity. (4) Sufficient data on detailed curve characteristics were lacking. In cases of severe deformity—such as curves exceeding 90°, significant rotation, or high stiffness—implants with greater mechanical stability may be needed to achieve effective correction and long-term integrity. Moreover, the selection of the UIV and LIV is crucial, as it directly affects not only the corrective capacity and construct stability but also the risk of junctional complications. However, most of the included studies did not provide detailed information on curve length, rigidity, or specific UIV/LIV selection strategies, which limited further analysis of the interaction between screw density and fusion-level configuration. This limitation highlights the need for future research to better define the relationships among curve characteristics, the fusion range, and the implant strategy to optimize individualized surgical planning in patients with AIS. (5) Follow-up periods varied across different articles. Three studies had a follow-up period of 1 year, which was not long enough. The remaining studies had follow-up periods of at least 2 years, but there are still differences among them.

## 5 Conclusion

Compared with high-density screws, low-density pedicle screws reduced the operative time and blood loss in patients who had undergone AIS surgery. Importantly, this low-density approach did not increase complication or revision rates. Low-density pedicle screws achieved similar Cobb angles and Cobb angle corrections and maintain thoracic kyphosis comparable to that of high-density screws at the final follow-up. The cost per level was lower for low-density pedicle screws.

## Data Availability

The original contributions presented in the study are included in the article/Supplementary Material, and further inquiries can be directed to the corresponding author.
